# Author Correction: Identifying antibiotics based on structural differences in the conserved allostery from mitochondrial heme-copper oxidases

**DOI:** 10.1038/s41467-022-35600-y

**Published:** 2022-12-21

**Authors:** Yuya Nishida, Sachiko Yanagisawa, Rikuri Morita, Hideki Shigematsu, Kyoko Shinzawa-Itoh, Hitomi Yuki, Satoshi Ogasawara, Ken Shimuta, Takashi Iwamoto, Chisa Nakabayashi, Waka Matsumura, Hisakazu Kato, Chai Gopalasingam, Takemasa Nagao, Tasneem Qaqorh, Yusuke Takahashi, Satoru Yamazaki, Katsumasa Kamiya, Ryuhei Harada, Nobuhiro Mizuno, Hideyuki Takahashi, Yukihiro Akeda, Makoto Ohnishi, Yoshikazu Ishii, Takashi Kumasaka, Takeshi Murata, Kazumasa Muramoto, Takehiko Tosha, Yoshitsugu Shiro, Teruki Honma, Yasuteru Shigeta, Minoru Kubo, Seiji Takashima, Yasunori Shintani

**Affiliations:** 1 Department of Molecular Pharmacology, National Cerebral and Cardiovascular Center, Suita, Osaka Japan; 2 Department of Medical Biochemistry, Osaka University Graduate School of Frontier Biological Science, Suita, Osaka Japan; 3 Graduate School of Science, University of Hyogo, Hyogo, Japan; 4 Center for Computational Sciences, University of Tsukuba, Tsukuba, Ibaraki Japan; 5 RIKEN SPring-8 Center, 1-1-1 Kouto, Sayo, Hyogo Japan; 6 RIKEN Center for Biosystems Dynamics Research, Yokohama, Kanagawa Japan; 7 Department of Chemistry, Graduate School of Science, Chiba University, Inage, Chiba Japan; 8 Department of Bacteriology I, National Institute of Infectious Diseases, Tokyo, Japan; 9 Antimicrobial Resistance Research Center, National Institute of Infectious Diseases, Tokyo, Japan; 10 Center for Basic Education Integrated Learning, Kanagawa Institute of Technology, Atsugi, Kanagawa Japan; 11 Protein Crystal Analysis Division, Japan Synchrotron Radiation Research Institute, SPring-8, Sayo, Hyogo Japan; 12 Department of Microbiology and Infectious Diseases, Toho University School of Medicine, Tokyo, Japan; 13 Present Address: Structural Biology Division, Japan Synchrotron Radiation Research Institute, SPring-8; Sayo, Hyogo, Japan

**Keywords:** Membrane proteins, Target identification, Structural biology, Antibiotics

Correction to: *Nature Communications* 10.1038/s41467-022-34771-y, published online 08 December 2022

In this article the affiliation ‘Present address: Structural Biology Division, Japan Synchrotron Radiation Research Institute, SPring-8; Sayo, Hyogo, Japan” for Hideki Shigematsu was missing.

The original article has been corrected.

