# Supplementary material for: Identifying antibiotics based on structural differences in the conserved allostery from mitochondrial heme-copper oxidases
Source: Nat Commun. 2022 Dec 8;13:7591. doi: 10.1038/s41467-022-34771-y (PMC9731990; doi:10.1038/s41467-022-34771-y)
Supplement: Supplementary file 1 — Supplementary Information [file 41467_2022_34771_MOESM1_ESM.pdf]

Supplementary Information

Conserved allostery buried inside mitochondrial heme-copper oxidases can generate novel antibiotics

Yuya Nishida[1,2], Sachiko Yanagisawa[3]†, Rikuri Morita[4]†, Hideki Shigematsu[5], Kyoko Shinzawa-Itoh[3], Hitomi Yuki[6], Satoshi Ogasawara[7], Ken Shimuta[8,9], Takashi Iwamoto[2], Chisa Nakabayashi[1,2], Waka Matsumura[3], Hisakazu Kato[2], Chai Gopalasingam[5], Takemasa Nagao[1], Tasneem Qaqorh[1,2], Yusuke Takahashi[1], Satoru Yamazaki[1], Katsumasa Kamiya[10], Ryuhei Harada[4], Nobuhiro Mizuno[11], Hideyuki Takahashi[8], Yukihiro Akeda[8], Makoto Ohnishi[8], Yoshikazu Ishii[12], Takashi Kumasaka[11], Takeshi Murata[7], Kazumasa Muramoto[3], Takehiko Tosha[5], Yoshitsugu Shiro[3], Teruki Honma[6], Yasuteru Shigeta[4], Minoru Kubo[3], Seiji Takashima[2], Yasunori Shintani[1,2]*

Supplementary Fig. 1 to 7

Supplementary Table 1 to 2

Supplementary Figure 1

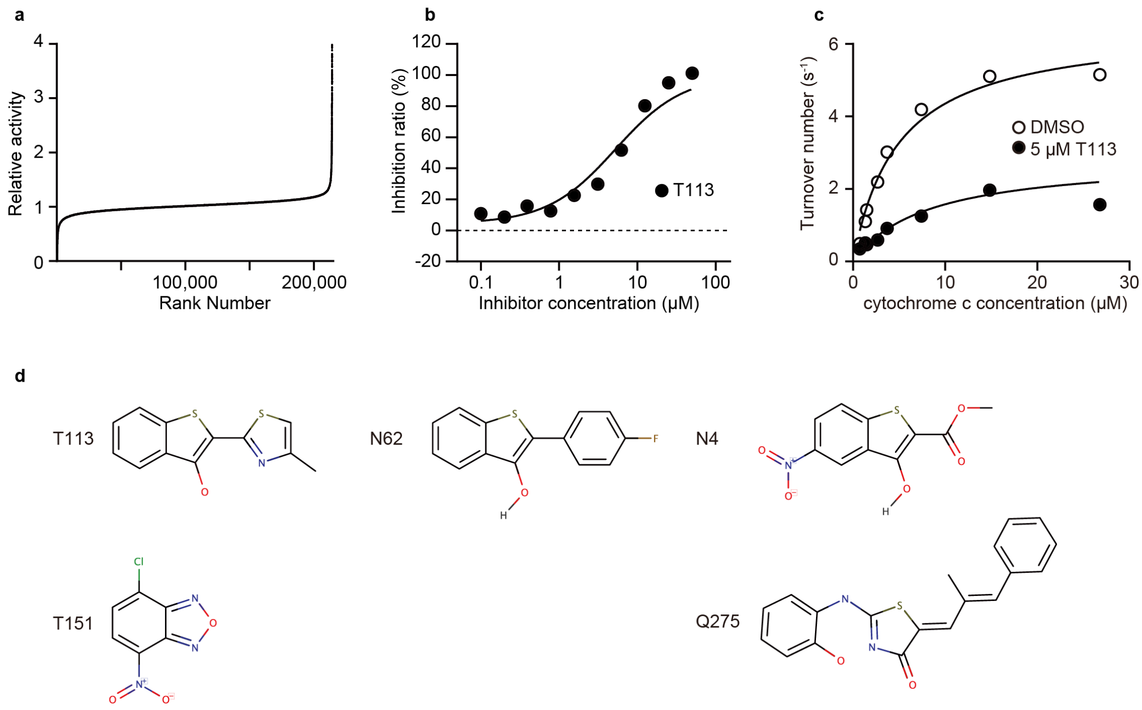

Supplementary Fig. 1 mtCcO inhibitors and kinetic and spectroscopic properties of T113.

a Primary screening data for inhibitory activity of 224,160 chemicals at 10 μM on mtCcO. Ratios of cytochrome *c* oxidization activity with chemicals to that with DMSO as negative control were calculated and sorted by the order. **b** Dose-dependent inhibition of T113 on mtCcO enzymatic activity. **c** Kinetic analysis of mtCcO with DMSO and 5 μM T113 molecule. Fitted lines are calculated by using Michaelis-Menten equation with non-competitive inhibition model. Data are presented as an average value of technical replicate. Reproducibility was confirmed by two independent experiments (**a-c**). **d** Chemical structure of T113, T151 (primary mtCcO inhibitor and queries used for establishing the custom compound library), N4 (*bo*$_3$ UqO inhibitor), N62 (mtCcO inhibitor used for resonance Raman spectroscopy), and Q275 (*bb*$_3$ qNOR inhibitor).

Supplementary Figure 2

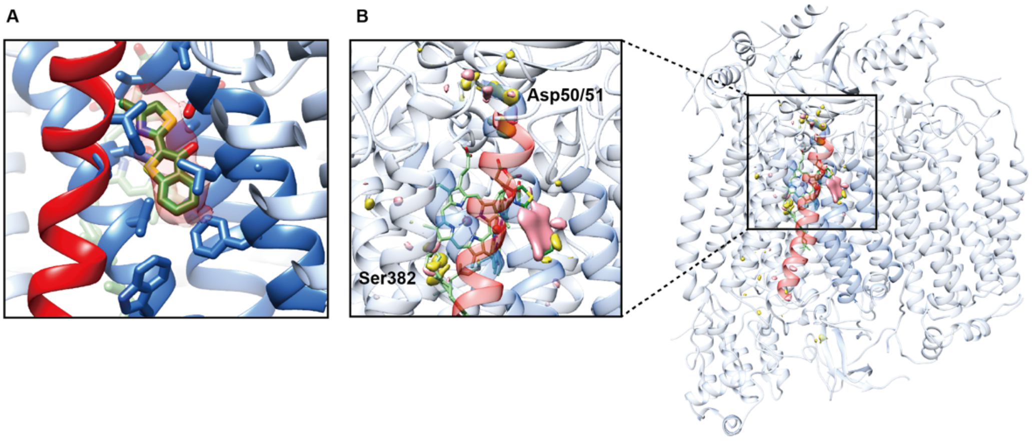

Supplementary Fig. 2 Co-crystallography of mtCcO with T113 revealed the allosteric site.

a T113 molecule in the allosteric site of mtCcO. The omit map ($2F_o$ - F_c), calculated by omitting T113 molecule and contoured at 1 σ, is shown around T113 molecule (red). **b** Structural differences between apo- and holo-structures. The F_o(T113) - F_o(DMSO) difference map, contoured at 5.5 σ, is shown with positive density (pink) and negative density (yellow). T113-derived density inside mtCcO shows the highest difference. Structural differences around Asp50/51, Ser382, and heme *a* are shown in the magnified box.

Supplementary Figure 3

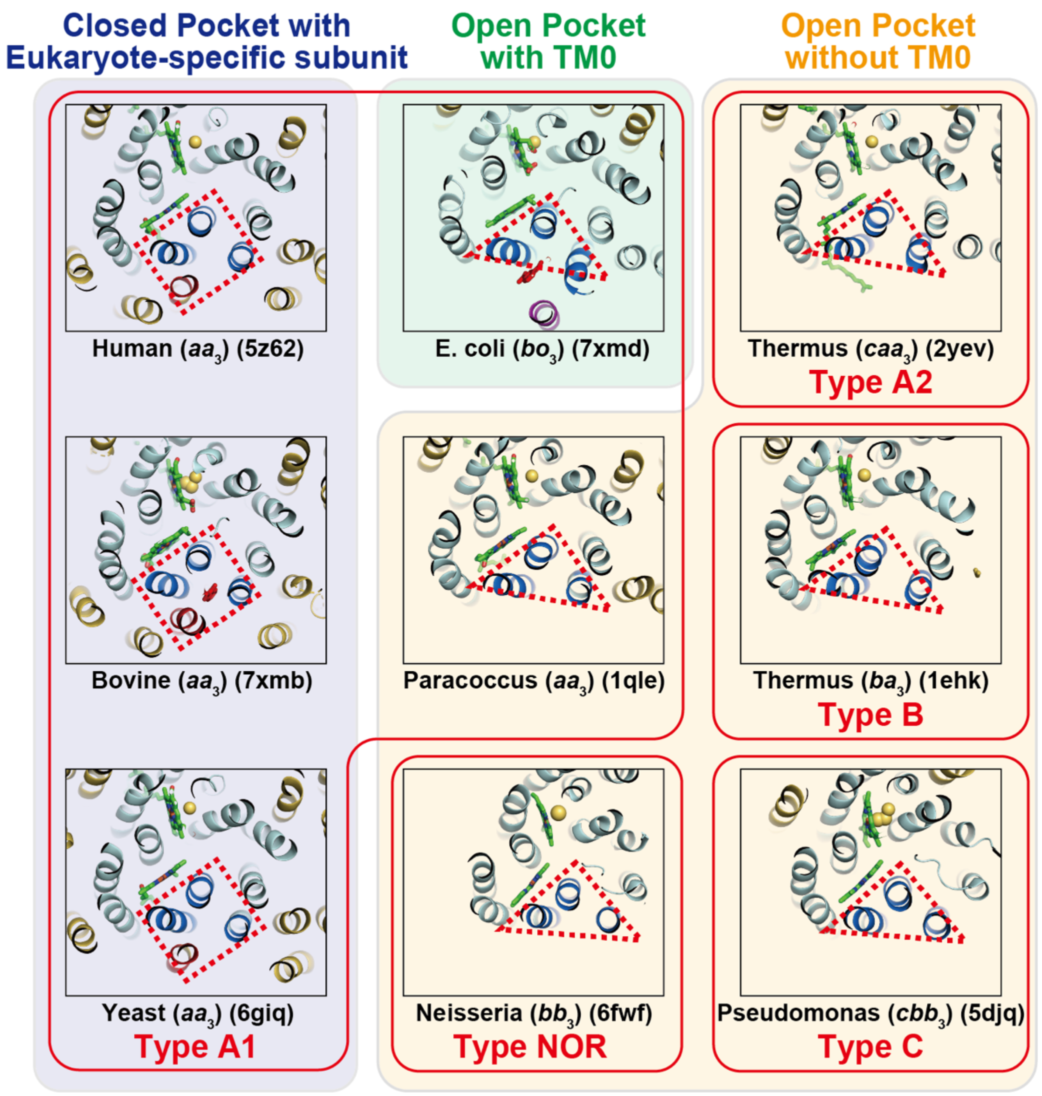

Supplementary Fig. 3 Comparison of the determined structures of HCOs.

The determined structures focused in conserved allosteric site (dot-lined red box) are shown. Closed pocket with eukaryote-specific subunit group, Open pocket with TM0 group, and Open pocket without TM0 group are shown in purple, green box, and yellow box, respectively. Heme type and PDB ID are labeled in parentheses. Color of the helices are as same as in Fig. 2.

Supplementary Figure 4

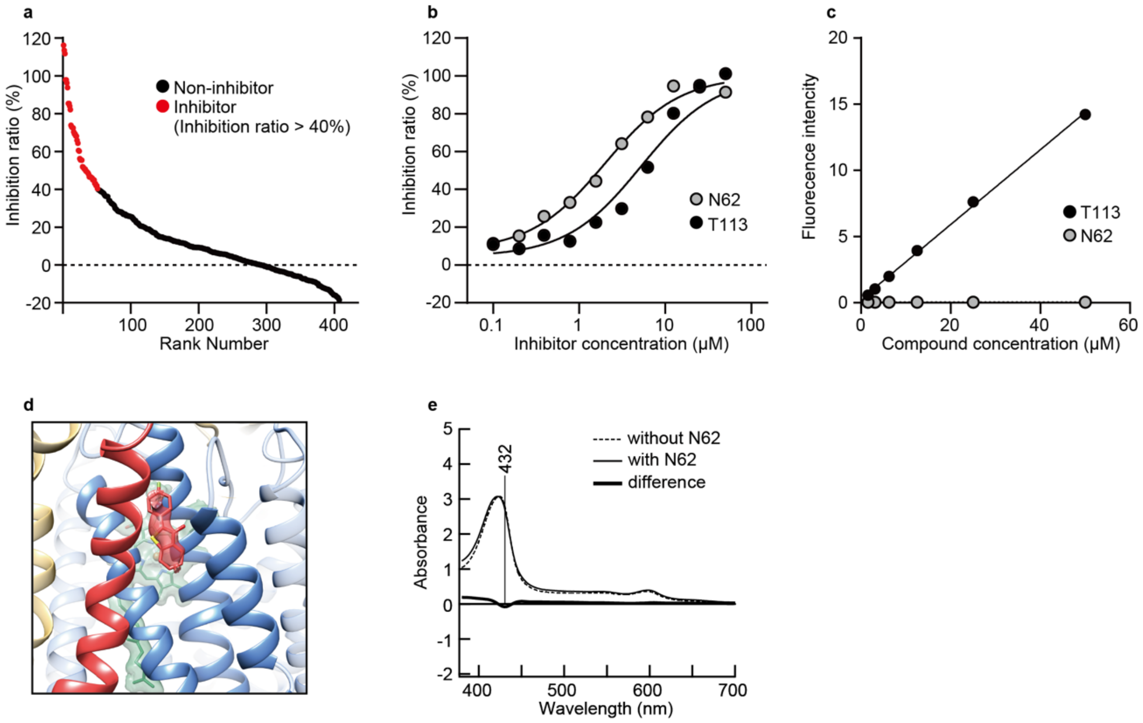

Supplementary Fig. 4 Screening for mtCcO inhibitor N62 and its spectroscopic properties.

a Screening of 434 chemicals at 50 μM against mtCcO. **b** Dose-dependent inhibition of T113 and N62 on mtCcO enzymatic activity. **c** Fluorescence intensity of T113 and N62 at 440 nm upon 410 nm excitation. Data are presented as an average value of technical replicate. Reproducibility was confirmed by two independent experiments (**a-c**). **d** N62 molecule in the allosteric site of mtCcO. The omit map ($2F_o - F_c$), calculated by omitting N62 molecule and contoured at 0.5 σ, is shown around N62 molecule (red) and heme *a* (green). **e** Absorption spectrum of oxidized mtCcO mixed with DMSO (without N62) and that with N62 and subtraction spectra are shown.

Supplementary Figure 5

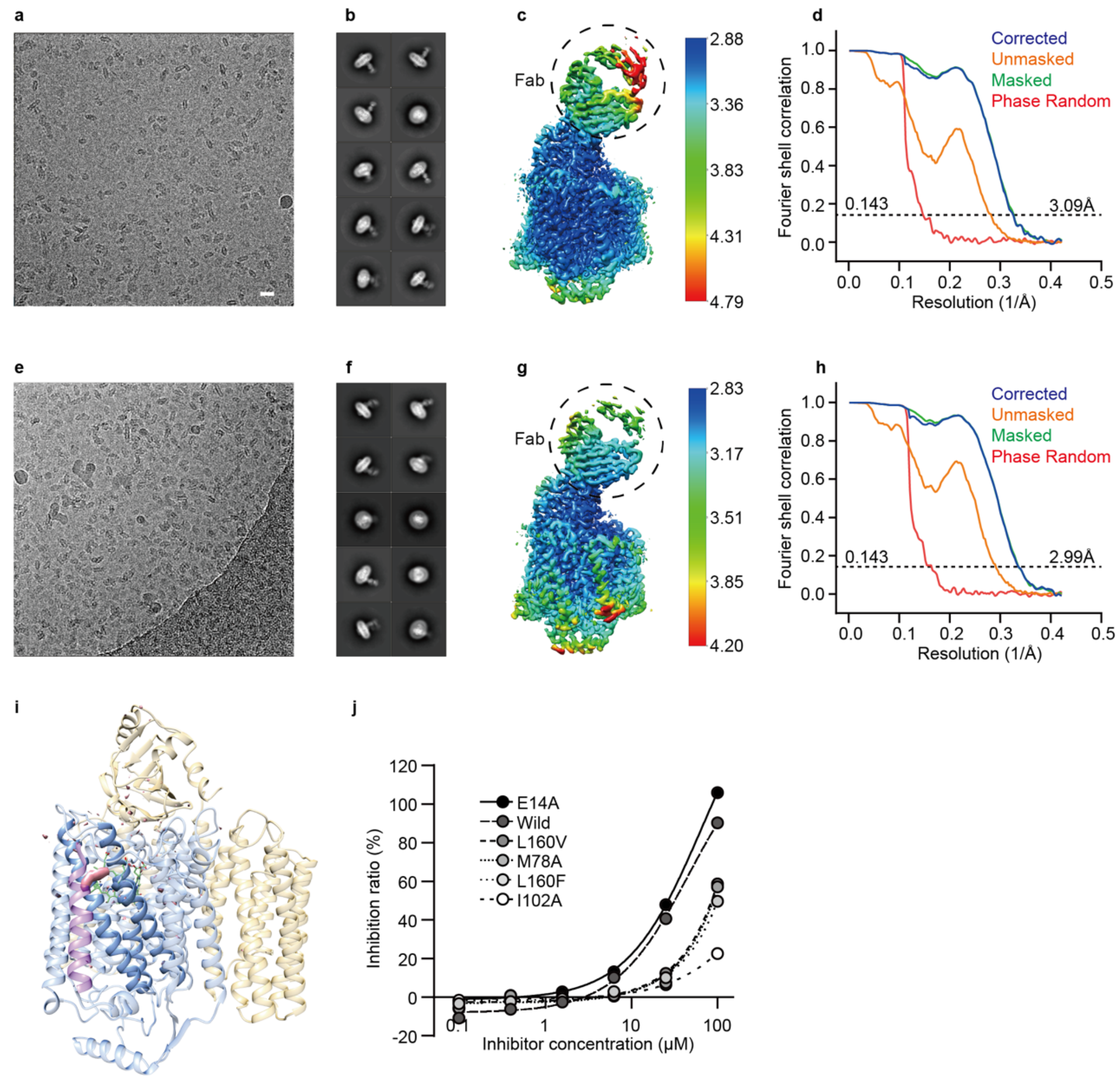

Supplementary Fig. 5 Cryo-EM analysis of apo- and holo-*bo*$_3$ UqO.

a and **e** Typical micrographs for apo-*bo*$_3$ UqO (**a**) and holo- (**e**). Entire micrographs are shown. Scale bars are 20 nm. (**b**) and (**f**) Two-dimensional class average for apo-*bo*$_3$ UqO (**b**) and holo- (**f**). (**c**) and (**g**) Overall views of the cryo-EM density for apo-*bo*$_3$ UqO (**c**) and holo- (**g**). The local resolution is indicated by difference color. There are few differences in local resolution around inhibitor. (**d**) and (**h**) The overall resolution is estimated to be 3.09 Å for apo- and 2.99 Å for holo- on the basis of the gold-standard FSC criteria of 0.143. **i** Structural differences between apo- and holo- structures. The

equal-volume 3D difference blobs between apo- and holo- cryo-EM density maps are shown with positive density (pink) and negative density (yellow). Three helices of subunit I surrounding the allosteric site are shown as dark blue, the other helices of subunit I as pale blue, TM0 of subunit I as purple, the other helices as yellow. **j** The effect of amino acid substitution of of *bo*3 UqO around the inhibitor. Met78, Ile102 and Leu160 are positioned in the allosteric site, and Glu14 is not neighboring to the allosteric site as a negative control. Data are presented as an average value of technical replicate. Reproducibility was confirmed by two independent experiments.

Supplementary Figure 6

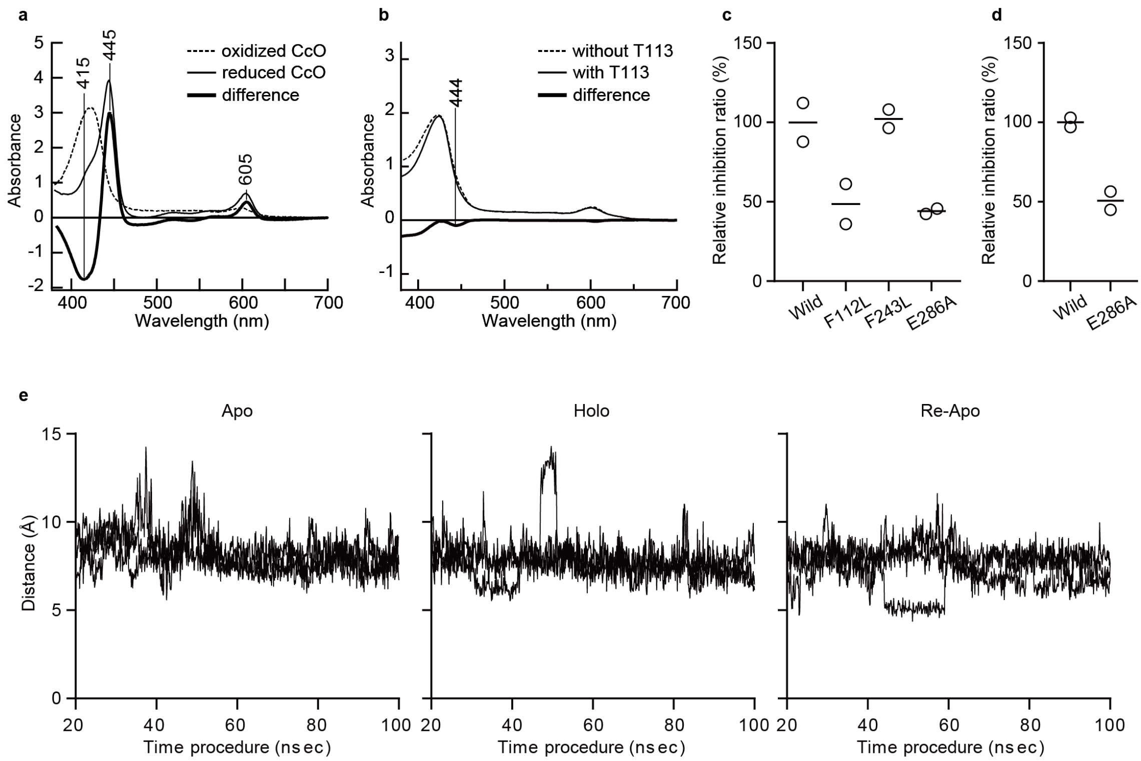

Supplementary Fig. 6 Mechanism of inhibition by HCO allosteric inhibitors.

a Absorption spectra of oxidized mtCcO and reduced mtCcO. Their difference spectrum is also shown. **b** Absorption spectra of oxidized mtCcO mixed with DMSO (without T113) and that with T113. Their difference spectrum is also shown. **c, d** Amino acid substitution in the oxygen channel of *bo*$_3$ UqO reduced the inhibitory effect of N4 (**c**) and N62 (**d**). Data are presented as an average value of technical replicate over two independent experiments. **e** MD simulation with the inhibitor did not cause the structural change around Asp50/51. Distribution histograms of the distance between Asp51-S441 in apo-MD, holo-MD and Re-apo MD.

Supplementary Figure 7

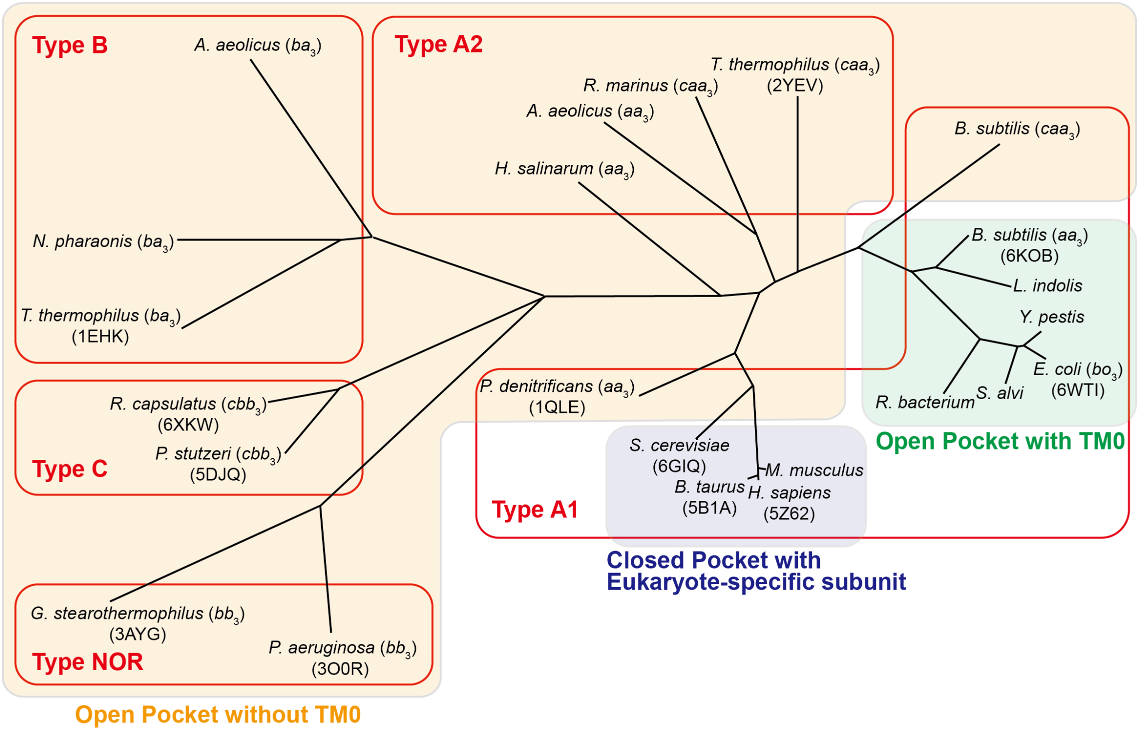

Supplementary Fig. 7 Unrooted radiation tree illustrating the phylogenic relationship between heme-copper oxidases (HCOs) subunit I.

Traditional classification of HCOs is shown as red line boxes. By the classification with types of the allosteric site, elongated chain (TM0) type, additional subunit type, and open pocket type are shown as a blue box, a green box, and yellow boxes, respectively. Heme type and PDB ID are labeled in parentheses.

	Apo (DMSO)	Holo (T113)
**Data Collection**		
Beamline	SPring8 BL26B1	SPring8 BL26B1
Wavelength (Å)	1.000	1.000
Space groups	$P2_12_12_1$	$P2_12_12_1$
Unit cell constants		
a, b, c (Å)	181.8, 203.58, 177.86	182.10, 204.34, 177.93
α, β, γ (°)	90, 90, 90	90, 90, 90
**Scaling**		
Resolution (Å)	29.97–2.20 (2.33–2.20)	29.98–2.20 (2.33–2.20)
Observed reflections	2,494,453 (379,988)	2,486,498 (385,342)
Independent reflection	644,464 (103,395)	648,641 (104,005)
Averaged redundancy	3.87 (3.68)	3.83 (3.71)
Completeness (%)	99.4 (99.2)	99.7 (98.9)
R_{merge}	6.9 (32.7)	6.4 (38.6)
R_{pim}	7.0 (38.5)	7.4 (45.2)
$CC_{1/2}$	99.8 (93.8)	99.8 (92.1)
$I/\sigma(I)$	15.96 (4.36)	14.80 (3.70)
**Refinement**		
Number of reflections in work set	611,550 (32,230)	616,024 (60,726)
Number of reflections in test set	60,978 (3,232)	32,427 (3,196)
R_{work}* (%)	17.99 (25.12)	17.06 (24.14)
R_{free}* (%)	20.83 (28.79)	19.74 (27.12)
Non-hydrogen atom numbers		
Total	33,576	33,766
Proteins	28,919	29,033
Water	2,487	2,495
Ligands	2,170	2,238
R.m.s. deviations		
Bonds (Å)	0.004	0.004
Angles (°)	0.857	0.905
Ramachandran statistics		
Favoured (%)	97.61	97.73
Allowed (%)	2.19	2.15
Outliers (%)	0.20	0.12
Clashscore	6.15	5.75

Average B-factor (Å2)	45.44	42.41
**PDB ID**	**7XMA**	**7XMB**

Supplementary Table 1 X-ray crystallography and refinement statics

Numbers in parentheses are given for the highest resolution shells.* R_{free} was calculated as the R_{work} for 5% of the reflections that were not included in the refinement.

	Apo (DMSO)	Holo (N4)
**Data Collection**		
Microscope	Glacios	Glacios
Camera	Gatan K2	Gatan K2
Magnification	45,000	45,000
Voltage (kV)	200	200
Dose (/eÅ^2)	50	50
Pixel size (Å)	0.889	0.889
Defocus range (μM)	-0.5–2.0	-0.5–2.0
Recorded movies	12,388	7,173
Final particle images	43,374	67,692
Accession number	EMD-33293	EMD-33294
**Image processing**		
Resolution ($FSC_{0.143}$) (Å)	3.09	2.99
Applied B-factor (Å)	-55.96	-52.64
**Refinement**		
Composition		
Total atom number	9,645	9,662
Protein residue number	1,197	1,197
Ligands	HEM 1, HEO 1, CU 1, PEE 2	HEM 1, HEO 1, XN4 1, CU 1, PEE 2
R.m.s. deviations		
Bonds (Å)	0.004	0.005
Angles (°)	0.682	0.697
Ramachandran statistics		
Favoured (%)	94.28	95.88
Allowed (%)	5.05	4.04
Outliers (%)	0.67	0.08
Average B-factor (Å^2)		
Protein	84.42	82.01
Ligand	81.72	79.09
Validation		
$FSC^{map\text{-}to\text{-}model}_{(0.5)}$ (Å)	3.1	3.0
$CC^{map\text{-}to\text{-}model}$	0.83	0.84
MolProbity score	2.36	2.09
Clashscore	11.55	10.97

**PDB ID**	**7XMC**	**7XMD**

Supplementary Table 2 Cryo-EM data collection and validation
