# Supplementary material for: Identifying antibiotics based on structural differences in the conserved allostery from mitochondrial heme-copper oxidases
Source: Nat Commun. 2022 Dec 8;13:7591. doi: 10.1038/s41467-022-34771-y (PMC9731990; doi:10.1038/s41467-022-34771-y)
Supplement: Supplementary file 3 — Reporting Summary [file 41467_2022_34771_MOESM3_ESM.pdf]

nature portfolio

Corresponding author(s): YASUNORI SHINTANI

Last updated by author(s): 28/Oct/2022

Reporting Summary

Nature Portfolio wishes to improve the reproducibility of the work that we publish. This form provides structure for consistency and transparency in reporting. For further information on Nature Portfolio policies, see our Editorial Policies and the Editorial Policy Checklist.

Statistics

For all statistical analyses, confirm that the following items are present in the figure legend, table legend, main text, or Methods section.

n/a	Confirmed	
☐	☒	The exact sample size (n) for each experimental group/condition, given as a discrete number and unit of measurement
☐	☒	A statement on whether measurements were taken from distinct samples or whether the same sample was measured repeatedly
☒	☐	The statistical test(s) used AND whether they are one- or two-sided<br>*Only common tests should be described solely by name; describe more complex techniques in the Methods section.*
☒	☐	A description of all covariates tested
☒	☐	A description of any assumptions or corrections, such as tests of normality and adjustment for multiple comparisons
☐	☒	A full description of the statistical parameters including central tendency (e.g. means) or other basic estimates (e.g. regression coefficient) AND variation (e.g. standard deviation) or associated estimates of uncertainty (e.g. confidence intervals)
☒	☐	For null hypothesis testing, the test statistic (e.g. F, t, r) with confidence intervals, effect sizes, degrees of freedom and P value noted<br>*Give P values as exact values whenever suitable.*
☒	☐	For Bayesian analysis, information on the choice of priors and Markov chain Monte Carlo settings
☒	☐	For hierarchical and complex designs, identification of the appropriate level for tests and full reporting of outcomes
☒	☐	Estimates of effect sizes (e.g. Cohen's d, Pearson's r), indicating how they were calculated

Our web collection on statistics for biologists contains articles on many of the points above.

Software and code

Policy information about availability of computer code

Data collection	X-ray experiment were carried out at SPring-8 beamlines BL26B1/B2. For CryoEM analysis, Glacios microscopes operated at 200 kV and equipped with Gatan K2 summit detector. The data were automatically acquired by the beam-image shift method using the SerialEM software. All the MD simulations were performed using the GROMACS (2019).
Data analysis	XDS (build=20160617~), COOT (0.8.9~), Chimera (1.11~), Relion (3.1~) , CTFFind (4.1.13~), MotionCor2 (1.3.1~), Phenix (1.11.1~), CAVER (3.0), SerialEM (3.7.6~), MOE (2016.08~), MOLREP (11.4.4~), CNS (1.4), REFMAC5 (5.8~), Schrödinger suite (2016-1), Glide (7.0), GROMACS (2019)

For manuscripts utilizing custom algorithms or software that are central to the research but not yet described in published literature, software must be made available to editors and reviewers. We strongly encourage code deposition in a community repository (e.g. GitHub). See the Nature Portfolio guidelines for submitting code & software for further information.

Data

Policy information about availability of data

All manuscripts must include a data availability statement. This statement should provide the following information, where applicable:

- Accession codes, unique identifiers, or web links for publicly available datasets
- A description of any restrictions on data availability
- For clinical datasets or third party data, please ensure that the statement adheres to our policy

Source data for Figure 2B, C, D, E, 3E, 4B, C, D, E, F, G, H, I, 5B, D, F, G, S1A, B, C, S4A, B, C, D, S5D, H, J, S6A, B, C, D are provided with the paper. The cryo-EM maps

have been deposited in the Electron Microscopy Data Bank (EMDB) under accession code EMD-33293 [https://www.ebi.ac.uk/pdbe/entry/emdb/EMD-33293] (apo-bo3 UqO) and EMD-33294 [https://www.ebi.ac.uk/pdbe/entry/emdb/EMD-33294] (holo-bo3 UqO). The coordinates have been in the RCSB Protein Data Bank (PDB) under accession code 7XMA [http://doi.org/10.2210/pdb7xma/pdb] (apo-mtCcO), 7XMB [http://doi.org/10.2210/pdb7xmb/pdb] (holo-mtCcO), 7XMC [http://doi.org/10.2210/pdb7xmc/pdb] (apo-bo3 UqO), 7XMD [http://doi.org/10.2210/pdb7xmd/pdb] (holo-bo3 UqO). 5B1A [http://doi.org/10.2210/pdb5b1a/pdb] was used for initial phase calculation in X-ray diffraction experiment. 3AG2 [http://doi.org/10.2210/pdb3ag2/pdb] was used for MD simulation. For Supplementary Fig. 7, 5Z62 [http://doi.org/10.2210/pdb5z62/pdb], 5B1A [http://doi.org/10.2210/pdb5b1a/pdb], 6GIQ [http://doi.org/10.2210/pdb6giq/pdb], 6KOB [http://doi.org/10.2210/pdb6kob/pdb], 6WTI [http://doi.org/10.2210/pdb6wti/pdb], 1QLE [http://doi.org/10.2210/pdb1qle/pdb], 2YEV [http://doi.org/10.2210/pdb2yev/pdb], 1EHK [http://doi.org/10.2210/pdb1ehk/pdb], 6XKW [http://doi.org/10.2210/pdb6xkw/pdb], 5DJQ [http://doi.org/10.2210/pdb5djq/pdb], 3AYG [http://doi.org/10.2210/pdb3ayg/pdb], 3O0R [http://doi.org/10.2210/pdb3o0r/pdb]. Any additional data supporting the findings of this study or information other than source data are available from the authors on reasonable request.

Human research participants

Policy information about studies involving human research participants and Sex and Gender in Research.

Reporting on sex and gender	N/A
Population characteristics	N/A
Recruitment	N/A
Ethics oversight	N/A

Note that full information on the approval of the study protocol must also be provided in the manuscript.

Field-specific reporting

Please select the one below that is the best fit for your research. If you are not sure, read the appropriate sections before making your selection.

☒ Life sciences ☐ Behavioural & social sciences ☐ Ecological, evolutionary & environmental sciences

For a reference copy of the document with all sections, see nature.com/documents/nr-reporting-summary-flat.pdf

Life sciences study design

All studies must disclose on these points even when the disclosure is negative.

Sample size	Sample size (the number of collected micrographs and particles) for single particle cryoEM analysis was determined from the experience of membrane protein structural analysis. Datasets of more than 5000 micrographs give a reasonable resolution of the structures that will be obtained. We used 12388 micrographs for EMD-33293 and 7173 for EMD-33294. Generally, we performed two technical duplicates for biochemical and microbiological experiments for each group/condition and confirmed its reproducibility by at least two independent experiments. We present the results with a clear-cut difference.
Data exclusions	Data were excluded when readings in wavelength had too much noise or were an outlier.
Replication	Each experiment was repeated at least two times with a minimum of n = 2 technical replicates. Only reproducible experiments with clear-cut difference are reported in this manuscript.
Randomization	Generally, no randomization is required for the experimental design for structural analysis or biochemical analysis of a single target protein. However, it is of note that particles are randomized during data processing steps in Relion 3.1 (randomization during 2D classification, randomized half sets during Relion 3D). Randomized half sets of particles are used in the final reconstruction steps.<br>Microbiological experiments usually do not require randomization because there are already too many microbes in experimental condition (> 1 x 108 cells).
Blinding	Structural and biochemical analysis was performed on a single enzyme sample. The experimental design did not require blinding approach to ensure robustness of structural and biochemical data.

Reporting for specific materials, systems and methods

We require information from authors about some types of materials, experimental systems and methods used in many studies. Here, indicate whether each material, system or method listed is relevant to your study. If you are not sure if a list item applies to your research, read the appropriate section before selecting a response.

Materials & experimental systems

n/a	Involved in the study
☐	☒ Antibodies
☐	☒ Eukaryotic cell lines
☒	☐ Palaeontology and archaeology
☒	☐ Animals and other organisms
☒	☐ Clinical data
☒	☐ Dual use research of concern

Methods

n/a	Involved in the study
☒	☐ ChIP-seq
☒	☐ Flow cytometry
☒	☐ MRI-based neuroimaging

Antibodies

Antibodies used	The antibody for cryoEM anlysis of bo3 UqO was generated by Dr Ogasawara
Validation	The binding of the antibody to bo3 UqO was confirmed by size exclusion chromatography.

Eukaryotic cell lines

Policy information about cell lines and Sex and Gender in Research

Cell line source(s)	C2C12 cells were obtained from ATCC (CRL-1722)
Authentication	The cells were authenticated originally by ATCC.
Mycoplasma contamination	The cell line was not tested for mycoplasma contamination in our laboratory.
Commonly misidentified lines (See ICLAC register)	No commonly misidentified cell lines was used in this study.
